# Role of Hydroxamate-Based Histone Deacetylase Inhibitors (Hb-HDACIs) in the Treatment of Solid Malignancies

**DOI:** 10.3390/cancers5030919

**Published:** 2013-07-25

**Authors:** Antonino Grassadonia, Pasquale Cioffi, Felice Simiele, Laura Iezzi, Marinella Zilli, Clara Natoli

**Affiliations:** 1 Department of Experimental and Clinical Sciences, University ’G. d’Annunzio’, I-66013 Chieti, Italy; E-Mail: grassa@unich.it; 2 Hospital Pharmacy, “SS. Annunziata” Hospital, I-66013 Chieti, Italy; E-Mails: cioffip@gmail.com (P.C.); felicesimiele@yahoo.it (F.S.); 3 Oncology Department, “SS. Annunziata” Hospital, I-66013 Chieti, Italy; E-Mails: donnalaura@hotmail.com (L.I.); marizilli@gmail.com (M.Z.)

**Keywords:** hydroxamate-based histone deacetylase inhibitors, histone deacetylase inhibitors, vorinostat, belinostat, panobinostat, pracinostat, abexinostat, resminostat

## Abstract

Hydroxamate-based histone deacetylase inhibitors (Hb-HDACIs), such as vorinostat, belinostat and panobinostat, have been previously shown to have a wide range of activity in hematologic malignancies such as cutaneous T-cell lymphoma and multiple myeloma. Recent data show that they synergize with a variety of cytotoxic and molecular targeted agents in many different solid tumors, including breast, prostate, pancreatic, lung and ovarian cancer. Hb-HDACIs have a quite good toxicity profile and are now being tested in phase I and II clinical trials in solid tumors with promising results in selected neoplasms, such as hepatocarcinoma. This review will focus on their clinical activity and safety in patients with advanced solid neoplasms.

## 1. Introduction

Over the past decade, considerable research interest has been focused on developing innovative drugs able to target specific mechanisms strictly related to cancer initiation and progression. The major aim of this novel approach has been to produce anticancer agents with specific selectivity for cancer cells in order to obtain more effective treatments with less toxicities than classical approaches, such as chemotherapy and radiotherapy. This has been made possible by the extraordinary progress in the understanding of molecular pathways involved in cancer development, including those regulating the cellular epigenome in hematological and solid tumor malignancies [1]*.* Epigenetics refers to the heritable changes in gene expression caused by complex mechanisms regulating histone modification and chromatin remodeling, DNA methylation, loss of imprinting, and microRNAs interference, without changes in the DNA nucleotide sequence [2,3]. Dysregulation of these pathways has been related to the development of many different pathologies, including cellular aging and cancer [2,4]*.* A great deal of attention has been devoted in the last years to the study of histone acetylation, one of the best described epigenetic process, which represents a key regulatory mechanism regulating gene expression. Essentially, increased acetylation of histones is associated with an increased transcriptional activity, whereas deacetylation is associated with silencing of gene expression [5]. Histone deacetylases (HDACs) are a class of enzymes that influence gene expression by altering the acetylation status of nucleosomal histones as well as of a number of non-histone proteins the regulation of multiple cell pathways [6]. The HDAC protein family consists of 18 members and is divided into four classes based on size, cellular localization, number of catalytic active sites, homology to yeast HDAC proteins and susceptibility to different inhibitors [7,8]. Classes I, II, and IV are zinc (Zn^2+^)-dependent enzymes [9], while class III HDACs, or sirtuins, do not contain zinc and require nicotinamide adenine dinucleotide (NAD^+^) for their catalytic activity [10]. The Zn^2+^-dependent HDACs have a tube-like catalytic pocket with a Zn^2+^ at its end [11].

HDACs are over-expressed in several solid tumors, making them an attractive target of anti-cancer drugs [12]. Altered HDAC activity has been associated with a variety of cancers, including ovarian, gastric, lung, breast, pancreatic, colorectal and prostate cancer [13,14,15,16,17,18,19]. New drug discovery programs using a wide range of procedures, including structure-based drug design and high-throughput screening, has allowed to identify multiple categories of HDAC inhibitors (HDACIs).

### 1.1. Rational for Combining HDACIs with Other Anticancer Agents

Compared to normal cells, transformed cells are particularly sensitive to HDAC inhibition, indicating a pivotal role for HDACs on maintenance of the neoplastic phenotype [20,21,22]. These enzymes seem to confer a survival advantage to cancer cells by regulating the expression of genes involved in growth, differentiation and apoptosis [23]. For this reason, agents able to interfere with cell growth and survival are potential enhancer of the HDACIs anticancer activity.

According to this general assumption, most of the chemotherapeutic and targeted agents used for cancer treatment might synergize with HDACIs. Actually, experimental evidences support the ability of HDACIs to increase the anticancer activities of a plethora of agents, both *in vitro* and *in vivo* [24]. However, the mechanisms underlying the additive or synergistic effect of the combinations remain in many cases unknown. In most clinical trials the rationale for the combination is based on the preclinical anticancer activity of the compounds, but the specific molecular mechanism is usually not described [25].

In recent years, the greater understanding of the molecular alterations involved in cell transformation and the knowledge of the mechanisms of action of many anticancer agents, including HDACIs, have allowed a better characterization of the pathways involved in the synergism observed when some HDACIs are combined with other agents. For example, the recognized ability of HDACIs to affect the expression of genes involved in DNA-damage response is probably the cause of their synergism with cytotoxic agents that specifically determine DNA-damage, such as antimetabolites, platinum derivatives, alchilant agents and topoisomerase II inhibitors [26,27]. The synergism of HDACIs with taxanes might be explained by an increased microtubules stabilization due to the inhibition of HDAC-6, enzyme responsible for α-tubulin deacetylation [28]. Other compounds that synergize with HDACIs are the proteosome inhibitors such as bortezomib, marizomib, and carfilzomib [29]. The proteosome system is widely utilized by cancer cells to degrade the excess of misfolded proteins accumulated inside the cells during their rapid turnover, a process indispensable for cancer cell survival. In case of hampered proteosome activity, such as in presence of bortezomib, cells activate the formation of aggresome, an escape pathway that allows the degradation of misfolded proteins and that depends on the HDAC6-mediated deacetylation of α-tubulin [30]. The synergism between bortezomib and HDACIs is, therefore, due to the simultaneous inhibition of proteosome and aggresome, a dual block that determines intracellular accumulation of misfolded proteins and, as a consequence, cell apoptosis [31].

Another well described anticancer effect of HDACIs is the inhibition of angiogenesis, resulting from the downregulation of genes involved in angiogenesis, such as the vascular endothelial growth factor (VEGF) and the endothelial nitric oxide synthase (eNOS) [32,33,34]. This effect is the rationale for the association of HDACIs with antiangiogenetic agents, such as bevacizumab [35].

### 1.2. Structural Requirements of Hb-HDACIs

**Figure 1:**
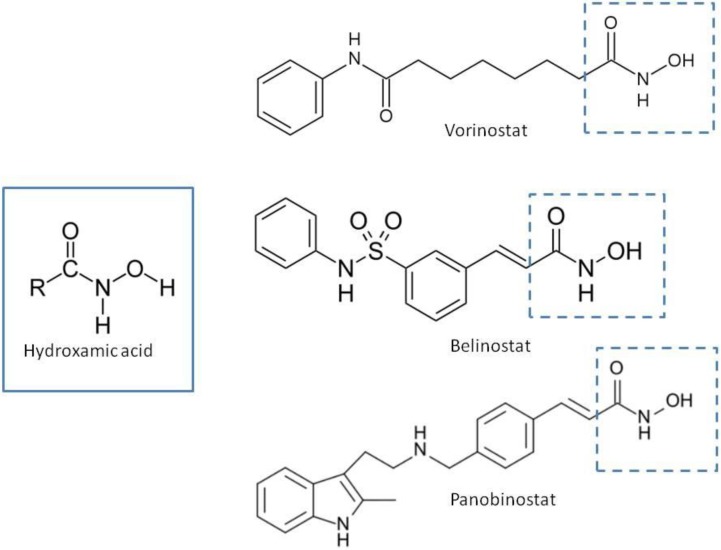
Chemical structures of Hb-HDACIs. Three different Hb-HDACIs, vorinostat, belinostat and panobinostat, are depicted in their planar formulae. The hydroxamic acid group, which characterizes this class of HDACIs, is represented in the square. It is responsible for the binding of these compounds to the zinc ion located into the catalytic domain of the enzyme.

Natural and synthetic HDACIs can be structurally divided into four major structural classes: hydroxamic acids (Hb-HDACIs), small molecular weight carboxylates, benzamides and cyclic peptides [36]. Hb-HDACIs typically contain a metal-binding moiety represented by the hydroxamic acid group (Figure 1).

**Figure 2:**
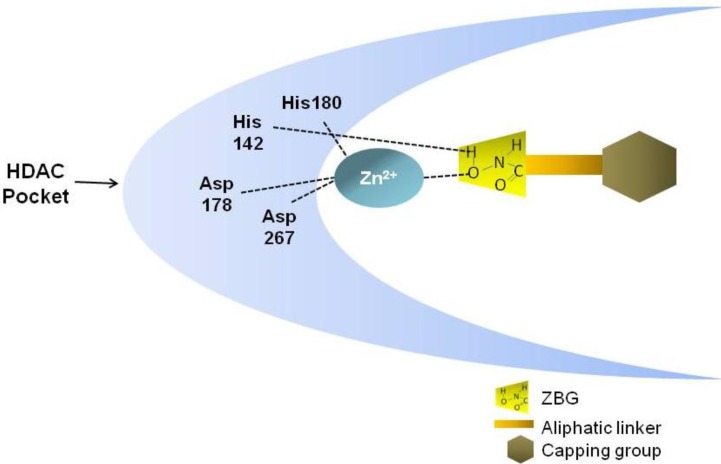
General structure of Hb-HDACI and its interaction with the HDAC enzyme. Hb-HDACIs contain three distinct structural motifs: a zinc binding group represented by the hydroxamic acid, an aliphatic linker that occupies the cavity of the HDAC enzyme, and a surface recognition cap group. The hydroxamic acid interacts with the zinc ion and the histidine 142 in the HDAC pocket inhibiting the enzyme activity.

The general structure of these compounds consists of three structural motifs that differently interact with the catalytic pocket of the HDAC enzyme: the hydroxamic acid group able to chelate the Zn^2+^ metal ions, an aliphatic linker, and a capping group that interacts with the residues at the entrance of the active site (Figure 2) [37]. By inserting in the HDAC catalytic pocket, Hb-HDACIs block the substrate access to the enzyme, inhibiting its activity with the consequence of an accumulation of acetylated histone and nonhistone proteins [38].

The Hb-HDACIs such as abexinostat (PCI-24781/CRA-024781), belinostat (PXD101), CHR-2845, CHR-3996, dacinostat (NVPLAQ824), givinostat (ITF2357), panobinostat (LBH-589), pracinostat (SB939), resminostat (4SC AG), trichostatin A and vorinostat (SAHA) can directly inhibit the HDAC enzyme by docking in its catalytic pocket, while others HDACIs, such as the cyclic tetrapeptide romidepsin (a non-Hb-HDACI), are prodrugs that require to be converted in a reduced intermediate to be active [39]. The compounds belonging to the class Hb-HDACIs and investigated in clinical trials are listed in Table 1. These agents show different toxicity profiles in solid tumors and have been administered by different routes (Table 2).

Several clinical trials have been completed and many others are ongoing using Hb-HDACIs in patients with different haematological malignancies, leading to approval of vorinostat on October 6, 2006 by the U.S. Food and Drug Administration (FDA) for the treatment of advanced forms of cutaneous T-cell lymphoma (CTCL) that have failed multiple other systemic treatment options [40,41,42,43]. Among Hb-HDACIs reported in literature [44,45], here we will focus only on those which are currently under investigation in phase I and phase II clinical trials for solid tumors, such as vorinostat, belinostat, panobinostat and other novel “second-generation” molecules, such as pracinostat, abexinostat, JNJ-26481585, dacinostat, and resminostat, which show promising clinical activity in this field.

**Table 1:** Hb-HDACIs in clinical trials for cancer treatment. The hydroxamic acid group, which characterizes this class of HDACIs, is represented in the red dotted square.

Hb-HDACIs	Structure	Chemical name	Formula	Molecular mass
Vorinostat	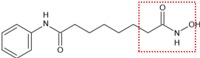	*N*-hydroxy-*N*'-phenyl-octanediamide	C_14_H_20_N_2_O_3_	264.32 g/mol
Belinostat (PXD101)	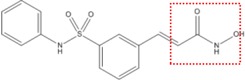	(2*E*)-*N*-Hydroxy-3-[3-(phenylsulfamoyl) phenyl]prop-2-enamide	C_15_H_14_N_2_O_4_S	318.35 g/mol
Panobinostat (LBH589)	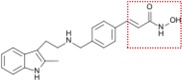	(2*E*)-*N*-hydroxy-3-[4-({[2-(2-methyl-1*H*-indol-3l)ethyl]amino}methyl) phenyl]acrylamide	C_21_H_23_N_3_O_2_	349.42 g/mol
Pracinostat (SB939)	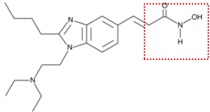	(E)-3-(2-butyl-1-(2-(diethylamino)ethyl)-1*H*-benzo[d]imidazol-5-yl)-*N*-hydroxyacrylamide	C_20_H_30_N_4_O_2_	358.48 g/mol
Abexinostat (PCI-24781 /CRA-024781)	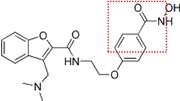	3-[(Dimethylamino) methyl]-*N*-{2-[4-(hydroxycarbamoyl) phenoxy]ethyl}-1-benzofuran-2-carboxamide	C_21_H_23_N_3_O_5_	397.42 g/mol
JNJ-26481585	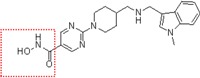	*N*-hydroxy-2-(4-(((1-methyl-1H-indol-3-yl)methylamino) methyl)piperidin-1-yl) pyrimidine-5-carboxamide	C_21_H_26_N_6_O_2_	394.47 g/mol
Dacinostat (LAQ824)	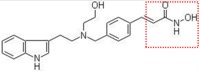	(*E*)-3-(4-(((2-(1H-indol-3-yl) ethyl)(2-hydroxyethyl)amino) methyl)phenyl)-*N*-hydroxyacrylamide	C_22_H_25_N_3_O_3_	379.45 g/mol
Resminostat (RAS2410/4SC-201)	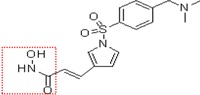	(*E*)-3-(1-((4-((dimethyl amino) methyl)phenyl)sulfonyl)-1*H*-pyrrol-3-yl)-*N*-hydroxyacrylamide	C_16_H_19_N_3_O_4_S	349.10 g/mol
CHR-3996	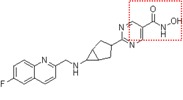	2-(6-(((6-fluoroquinolin-2-yl) methyl)amino) bicycle [3,1,0]hexan-3-yl)-*N*-hydroxypy rimidine-5-carboxamide	C_21_H_20_FN_5_O_2_	393.16 g/mol

**Table 2:** Hb-HDACIs in the clinic.

HDACIs	Routes of administration	Side effects	FDA approval
Vorinostat	Oral	Anorexia, fatigue, dehydration, diarrhea, and myelosuppression	Cutaneous T-cell lymphoma
Belinostat (PXD101)	Oral, i.v.	Lethargy/fatigue, nausea and vomiting	Granted orphan drug and fast track designations for relapsed or refractory peripheral T-cell lymphoma
Panobinostat (LBH589)	Oral, i.v	Fatigue, nausea, diarrhea and myelosuppression	Not approved
Pracinostat (SB939)	Oral	Fatigue, nausea, vomiting, anorexia and diarrhoea	Not approved
Abexinostat (PCI-24781/ CRA-024781)	Oral, i.v.	Under evaluation in clinical trials	Not approved
JNJ-26481585	Oral	Under evaluation in clinical trials	Not approved
Dacinostat (LAQ824)	i.v.	Nausea, vomiting and fatigue	Not approved
Resminostat (RAS2410/4SC-201)	Oral	Under evaluation in clinical trials	Granted orphan drug designation in relapsed/refractory Hodgkin’s lymphoma and hepatocellular carcinoma
CHR-3996	Oral	Thrombocytopenia, fatigue, increase of plasma creatinine and atrial fibrillation	Not approved

Abbreviations: FDA, United States Food and Drug Administration; i.v., intravenous.

## 2. Vorinostat in Solid Tumors

The discovery of hexamethylene bisacetamide (HMBA), a polar compound able to induce differentiation of transformed cells [39,46], and the attempt to overcome its severe side effects [46,47], especially thrombocytopenia, have brought to the development of vorinostat (SAHA), a second-generation compound endowed with greater potency (about 2,000-fold) and less toxicity than HMBA. This new Hb-HDACI has been tested in phase I/II clinical trials either as single agent or in combination therapy.

### 2.1. Vorinostat as a Single Agent

Oral vorinostat administered as 200 mg twice daily or 400 mg daily was a well-tolerated agent in phase I trials [48,49]. Pharmacokinetic analysis shows that the bioavailability of vorinostat ranges from 34.9% to 52.3% (only slightly improved with food), with a half-life of 91–127 minutes and an inhibition time of HDAC ≥ 10 hours [48]. Predominant toxicities of vorinostat include anorexia, fatigue, dehydration, diarrhea, and myelosuppression. It has also been suggested that the approved dose of vorinostat (single 400 mg p.o. daily dose) should be reduced in patients with hepatic dysfunction [49].

In the last few years, the clinical activity of Hb-HDACIs has been mainly tested in hematological malignancies with few studies testing their activity in solid tumors. In phase II trials (Table 3), no responses to oral vorinostat have been observed in patients with relapsed or refractory non small cell lung (NSCLC), colorectal, prostate, head and neck cancer [8,50,51,52,53,54]. A modest clinical activity of vorinostat has been reported in ovarian cancer, primary peritoneal carcinoma and recurrent glioblastoma multiforme [8,55,56]. In these phase II trials, vorinostat was well tolerated: anorexia, fatigue, nausea and vomiting, diarrhea, anemia and thrombocytopenia were frequently observed, but none of these drug-related toxicities was severe.

**Table 3:** Vorinostat in published phase II clinical trials in solid tumors.

Disease	Regimen	No. pts.	PFS	Efficacy	Ref.
Metastatic head and neck cancer	*Vorinostat:* 400 mg orally daily	13		SD 2 pts. OR 0%	[51]
Metastatic breast cancer	*Vorinostat:* 400 mg orally daily	14		SD 4 pts	[52]
Relapsed NSCLC	*Vorinostat*: 400 mg or 300 mg once daily on days 1–14 of the 21 day cycle	16	Median: 2.3 m.	SD 57%	[53]
Metastatic prostate cancer	*Vorinostat:* 400 mg orally daily	27	Median: 2.8 m.	SD 2 pts.	[54]
Ovarian or primary peritoneal carcinoma	*Vorinostat:* 400 mg orally daily	27		PR 1 pt.	[55]
Recurrent GBM	*Vorinostat*: 200 mg p.o.twice a day for 14 days, followed by a 7-day rest period	66	6-m.-PFS: 9 of the first 52 patients	OR 2 pts.	[56]
Metastatic colorectal cancer	*Vorinostat:* 800 or 1,400 mg/day once a day × 3 days, every 2 weeks *+5-FU:* preceded by leucovorin, at 400 mg/m^2^ followed by a 46 h infusion at 2.400 mg/m^2^ on days 2–3	58	PFS rate did not reach the threshold of 27 out of 43 patients	PR 1 pt.	[57]
Untreated stage IIIB or IV NSCLC	*Vorinostat*: 400 mg or Placebo on days 1 through 14 of each 3-week cycle +*Carboplatin*: AUC 6 + *Paclitaxel*: 200 mg/m^2^, both on day 3 of each 21-day cycle	94	Median: *Vorinostat*: 6.0 m. *Placebo*: 4.1 m. *P =* ns	*RR Vorinostat*: 34% *Placebo*: 12.5% *P* = 02	[58]
Advanced thyroid cancer	*Vorinostat:* 400 mg orally daily	19		SD 56% OR 0	[59]
Recurrent GBM	*Vorinostat:* 400 mg daily for 14 days of a 21-day cycle + *Bortezomib*: 1.3 mg/m2 intravenously on days 1,4, 8, and 11 of a 21-day cycle	37	Median: 1.5 m.	PR 1 pt.	[60]

Abbreviations: 5-FU, 5-fluorouracil; 6-m.-PFS, progression free survival at 6 months; AUC, area under the curve; CB, clinical benefit; GBM, glioblastoma multiforme; LV, leucovorin; m., months; MBC, metastatic breast cancer; No., number; NSCLC, non small cell lung cancer; OR, objective response; PFS, progression free survival; PR, partial response; pts., patients; Ref. , references; RR, objective response rate.

### 2.2. Vorinostat in Combination Therapy

Even if vorinostat demonstrated a low clinical activity in solid tumors, in consideration of its acceptable safety profile and the oral route of administration, further studies were carried out to investigate this drug in association with other antineoplastic agents in patients affected by different types of advanced solid tumors. Vorinostat has been tested in combination with 5-FU, leucovorin (LV) with or without oxaliplatin in patients with metastatic colorectal cancer [61,62,63]. The rational of these association is based on the reported anticancer synergisms between vorinostat and both 5-FU and oxaliplatin *in vitro* and *in vivo* colorectal cancer models [63,64]. However, the combination of these drugs did not allow to obtain significant improvements in response rate or time to disease progression, the best results being represented by disease stabilization in some patients [61,62,63].

The combination of vorinostat with docetaxel, in patients with advanced or recurrent cancer, resulted in a very high toxicity without any clinical activity, leading to an early closure of the trial [28]. Similarly, vorinostat, in combination with weekly doxorubicin in solid tumors showed a minimal activity at the expenses of a high incidence of hematological toxicity and thromboembolic events [65]. On the other hand, oral vorinostat at 200 mg for 7 days in combination with weekly vinorelbine at 25 mg/m^2^ was a well tolerated regimen, even if two cases of grade 3 hyperglycemia were observed at the first dose level [66]. More promising results have been obtained in patients with metastatic NSCLC and breast cancer [67,68]. In preclinical models, vorinostat, when co-administered with platinum agents, has been shown to increase platinum adduct formation by relaxing the chromatin structure, thus allowing a more efficient anticancer activity of the alkylating agents [69]. Vorinostat has also been reported to synergize with taxanes through the inhibition of HDAC-6, responsible of α-tubulin de-acetylation [70]. These mechanisms could lead to more stable microtubules, resulting in the arrest of cell division [28]. In NSCLC, vorinostat in combination with carboplatin and paclitaxel allowed to obtain a response rate of 34% as compared to 12.5% of placebo, with the vorinostat arm showing a trend toward a longer progression-free survival (6.0 months *vs.* 4.1 months) and overall survival (13.0 months *vs.* 9.7 months) [58]. However, a higher incidence of toxicity, including nausea/vomiting, fatigue, hyponatremia, and thrombocytopenia in the vorinostat arm, prompted 26% of patients to discontinue therapy [58].

Oral vorinostat at 400 mg daily for 3 out of 4 weeks and oral tamoxifen at 20 mg daily, in patients with endocrine-resistant advanced breast cancer, obtained an objective response rate of 19%, a clinical benefit rate of 40%, and a median time to disease progression of 10.3 months [68]. The favorable toxicity profile of this combination allowed up to 2 years treatment for responding patients [58].

Good results are coming from trials testing vorinostat in combination with chemotherapy and targeted therapy such as bevacizumab, the anti-vascular endothelia growth factor monoclonal antibody [71]. In patients with metastatic breast cancer, vorinostat (200 or 300 mg p.o. BID) on days 1–3, 8–10, and 15–17, plus paclitaxel (90 mg/m^2^) on days 2, 9, 16, and bevacizumab (10 mg/kg) on days 2 and 16 every 28 days, allowed to obtain a response rate of 55%, with only an increased incidence of diarrhea with respect to the expected toxicity profile of paclitaxel and bevacizumab [72].

Vorinostat has also been tested in combination with bevacizumab and CPT-11 in patient with recurrent glioblastoma [73]. Dose-limiting toxicities were represented by mucositis, fatigue and diarrhea, with a trend for better progression free survival and overall survival for patients treated with the higher doses of vorinostat [73]. However, vorinostat in combination with the proteasome inhibitor bortezomib [74] had only a minimal clinical activity in the same category of patients [60]. Vorinostat was also combined with flavopiridol, an inhibitor of cyclin-dependent kinases [75]. In a phase I study, eight out of 34 patients with solid tumors refractory to standard therapy had stable disease for an average 5.5 months, with no objective responses being observed [76]. In another phase I study vorinostat was associated with the novel proteasome inhibitor marizomib in 22 patients with melanoma, pancreatic carcinoma or NSCLC. The combination was well tolerated with a stable disease observed in 61% of patients, including 39% with a minimal tumor size reduction [77].

## 3. Belinostat in Solid Tumors

Belinostat (PXD101) is a novel and potent class I and II Hb-HDACI, active at nanomolar concentrations [78,79]. Belinostat demonstrated a high antitumor activity both *in vitro* and *in vivo*, either alone or in combination with antineoplastic agents such as paclitaxel, docetaxel and carboplatin [79,80]. Belinostat, tested in phase II clinical trials for solid tumors, has shown an encouraging clinical activity (Table 4). The response rate and the safety profile of belinostat administered as a second line therapy in patient with recurrent or refractory malignant pleural mesothelioma was evaluated in a phase II study [81]. In this trial, among 13 patients evaluable for response assessment, only two patients had stabilization of disease, with no partial responses.

**Table 4:** Belinostat in published phase II clinical trials in solid tumors.

Disease	Regimen	No. pts.	PFS	Efficacy	Ref.
Recurrent or refractory malignant pleural mesothelioma	*Belinostat*: 1 g/m^2^ IV on days 1 to 5 of a 21-day cycle	13	Median: 1 m.	SD 2 pts.	[81]
Recurrent or refractory advanced thymic epithelial tumors	*Belinostat*: 1 g/m^2^ IV on days 1 to 5 of a 21-day cycle	41	6-m.- PFS: 46%	RR 8% CB 68%	[82]
Unresectable hepatocellular carcinoma	*Belinostat*: 1,400 mg/m^2^ per day, on days 1–5 every 3 weeks	54	Median: 2.64 m.	PR 2.4% SD 45.2%	[83]
Platinum resistant EOC and LMP ovarian tumors	*Belinostat*: 1 g/m^2^ IV on days 1 to 5 of a 21-day cycle	32	*EOC*: Median: 2.3 m. *LMP:* Median: 13.4 m.	*EOC:* SD 9/15 pts *LMP:* PR 2/12 pts	[84]
Previously treated ovarian, fallopian tube, or primary peritoneal carcinoma	*Belinostat*: 1g/m² IV daily for 5 days of a 21-day cycle *+Carboplatin*: AUC 5 on day three of 21-day cycles	29	Median: 3.3 m.	RR 7.4% CR 3.7% PR 3.7% SD 44.4%	[85]
Previously treated ovarian cancer	*Belinostat*: 1g/m² IV daily for 5 days of a 21-day cycle +*Carboplatin*: AUC 5 +*Paclitaxel*: 175 mg/m² both on day 3 of each 21-day cycle	35	6-m. PFS: 48%	RR 43%	[86]

Abbreviations: 6-m.-PFS, progression free survival at 6 months; AUC, area under the curve; CB, clinical benefit; CR, complete response; EOC, epithelial ovarian cancer; LMP, micropapillary; m., months; No., number; PFS, progression free survival; pts, patients; Ref., references; RR, objective response rate.

### 3.1. Belinostat as a Single Agent

In a phase I study, belinostat, administered i.v. on days 1 to 5 every 21 days and tested on 48 patients with advanced malignancies, showed a good tolerability profile with the maximum tolerated dose set at 1,000 mg/m^2^/d i.v. [87]. At this dose level, the most common adverse events were represented by lethargy/fatigue, nausea and vomiting. Interestingly, 39% of patients treated with belinostat achieved a stable disease and showed an increment of a caspase-dependent cleavage of cytokeratin 18 (a serum marker of apoptosis in epithelial tumors) during treatment [87,88]. Belinostat in monotherapy has also been tested in 41 patients with advanced thymic epithelial malignancies after a first line platinum chemotherapy regimen [82]. Interestingly, two patients affected by thymoma showed a partial response, 25 patients achieved a stable disease, while patients affected by thymic carcinoma did not respond to treatment. A phase I–II study in patients with unresectable hepatocellular carcinoma, belinostat at 1,400 mg/m^2^ per day, on days 1–5 every 3 weeks, allowed to obtain stable disease 45.2% of patients, with a median progression free survival of 2.64 months (95% CI, 1.55–3.17 months) [83]. Belinostat, at the standard dose of 1,000 mg/m^2^/d on days 1–5 of a 21 day cycle, was tested in patients with platinum resistant metastatic or recurrent epithelial ovarian cancer (EOC) and micropapillary/LMP ovarian tumors [84]. Generally, belinostat was very well tolerated with fatigue and nausea representing the most frequent side effects. Nine out of 15 patients with platinum resistant EOC had stable disease and among 12 patients with micropapillary/LMP tumors, two partial responses have been observed. Similar promising results have been obtained by the combination of belinostat and carboplatin in patients with platinum-resistant ovarian, fallopian tube, or primary peritoneal carcinoma: the overall response rate was 7.4%, with 44.4% of patients reaching stable disease [85].

### 3.2. Belinostat in Combination Therapy

Belinostat at the maximum dose of 1,000 mg/m^2^ in association with carboplatin and/or paclitaxel was a well tolerated regimen in patients affected by solid tumors, the most common toxicities being represented by neutropenia, thrombocytopenia and anemia, peripheral sensory neuropathy, fatigue, vomiting and myalgia [89]. In a phase II study carried out on 35 women with recurrent ovarian cancer, belinostat in combination with carboplatin and paclitaxel showed an overall response rate of 43% and a well tolerated profile [86].

## 4. Panobinostat in Solid Tumors

Panobinostat (LBH589) is a novel cinnamic hydroxamic acid class of HDACIs, formulated as oral capsule or i.v. solution [90]. Panobinostat improves its anticancer activity through the hyper-acetylation of histone and non-histone proteins thus modifying chromatin structure, gene expression, transcriptional activity and apoptosis [91,92].

### Efficacy of Panobinostat in Solid Tumor

Panobinostat is mainly metabolized by the human cytocrome P450 CYP3A4 and strong CYP3A4 inhibitors, such as ketoconazole, are able to increase panobinostat AUC by ~2-fold with a subsequent higher incidence of gastrointestinal adverse events [93].

In a phase I trial in patients affected by solid tumors or cutaneous T-cell lymphoma, oral panobinostat at 20 mg/m^2^, once daily on three alternating days of each week on a 28-day cycle, was considered as the maximum tolerated dose to be tested in phase II trials [94]. Dose limiting toxicities were represented by thrombocytopenia, anemia and, to a lesser extent, by diarrhea and nausea [94].

Panobinostat has also been tested in other phase I trials in combination with antineoplastic drugs, such as gemcitabine, docetaxel, paclitaxel, carboplatin, everolimus and bevacizumab [95,96,97,98]. In these trials oral panobinostat was used at different schedules: 30 mg three times per week every other week [35], 20 mg on three alternating days of weeks 1–2 every 21 days [95], 10 mg on three alternating days of weeks 1–2 every 21 days [98], or weekly [96,97]. Limited clinical activity has been shown by the association of panobinostat with paclitaxel and carboplatin [96], with gemcitabine [98] or with everolimus and bevacizumab [97]. In a phase I trial accruing patients with castration-resistant prostate cancer, panobinostat was administered as monotherapy (20 mg orally on three alternating days of weeks 1–2, every 4 weeks) or in combination with docetaxel 75 mg/m^2^ every 3 weeks plus prednisone 5 mg daily [95]. All 16 patients who received panobinostat monotherapy and two out of eight patients treated with panobinostat in combination with docetaxel had progressive disease [95]. The best promising clinical activity has been shown by the combination of panobinostat with bevacizumab in patients with recurrent high-grade glioma: among 12 enrolled patients, three patients had partial response and seven patients reached stable disease [35].

In phase II trials (Table 5), panobinostat did not show any significant clinical activity, either in monotherapy in patients with advanced refractory renal carcinoma [99], or in combination with bortezomib in patients with advanced refractory pancreatic cancer [100].

**Table 5:** Panobinostat in published phase II clinical trials in solid tumors.

Disease	Regimen	No. pts.	PFS	Efficacy	Ref.
Refractory metastatic renal cell carcinoma.	*Panobinostat*: 45 mg orally twice a week	20		OR 0	[99]
Previously treated advanced pancreatic cancer	*Panobinostat*: 20 mg orally three times weekly *+Bortezomib*: 1.3 mg/m^2^ IV twice weekly, both during the first two weeks, followed by a 9-day rest period.		Median: 2.1 m.	OR 0	[100]

Abbreviations: m., months; MBC, metastatic breast cancer; No., number; OR, objective response; PFS, progression free survival; pts, patients; Ref., references.

## 5. Other Hb-HDACIs in Solid Tumors

### 5.1. Pracinostat

Pracinostat (SB939) is a potent Hb-HDACI, able to inhibit HDACs class I, II, and IV, developed with the aim to increase the pharmacokinetic properties and anti-tumor activities of this class of drugs [101]. In two different phase I clinical trials performed in patients with advanced solid tumors, the recommended dose of pracinostat was fixed at 60 mg orally given either for five consecutive days every 2 weeks or 3 times a week for 3 weeks in a 4-week cycle [102,103]. Pracinostat showed a promising clinical acivity [102,103], and was well tolerated, with the most common side effects being represented by fatigue, nausea, vomiting, anorexia and diarrhoea.

### 5.2. Abexinostat

Abexinostat (PCI-24781/CRA-024781) is a Hb-HDACI with a high antitumor activity both *in vitro* and *in vivo* in human solid tumor xenografts [104]. Great interest has been devoted to the potential activity of abexinostat either alone or in combination with other anti-cancer agents in experimental models of soft tissue sarcomas [105,106,107], malignant peripheral nerve sheath tumors [108], glioblastoma [109] and gallbladder carcinoma [110]. The drug is currently tested in patients with refractory advanced solid tumor patient with promising clinical activity, but full data have not yet been published.

### 5.3. JNJ-26481585

JNJ-26481585 is an oral powerful pan-HDACI endowed with *in vitr*o antitumor activity against lung, breast, colon, prostate, brain, and ovarian cancer cell lines and *in vivo* as a single agent in xenografts tumor models [111]. Interestingly, JNJ-26481585 was more active in inhibiting cell proliferation of Ras mutant than Ras wild-type colorectal cancer cells [111].

### 5.4. Dacinostat

Dacinostat (LAQ824) is a water soluble synthetic cinnamyl Hb-HDACI, able to inhibit HDAC at less than 0.15 μM [112]. It has been shown to be active *in vitro* and *in vivo* against multiple human solid tumors [21,113,114,115,116,117,118,119,120]. In a phase I study in patients with advanced solid tumors, dacinostat was administered i.v. as a 3-h infusion on days 1, 2, and 3 every 21 days and the recommended dose was set from 24 to 72 mg/m^2^ [121]. The most common toxicities of dacinostat were similar to those of other HDACIs, and represented by nausea, vomiting and fatigue. The clinical activity of dacinostat was limited, with the best response represented by disease stabilization in three out of 39 patients enrolled in the study [121].

### 5.5. Resminostat

The oral pan-Hb-HDACI resminostat (RAS2410/4SC-201) [122] has been used in a phase I trial in patients with advanced refractory solid tumors [123]. Nausea, vomiting and fatigue represented the most common drug-related toxicities. The recommended dose for phase II evaluation was 600 mg once daily, on day 1–5 in a 14-day cycle [123]. Resminostat is currently being tested in phase I/II clinical trials in hepatocellular carcinoma in monotherapy or in combination with sorafenib (SHELTER trial) [124] and as a second-line treatment option in patients with KRAS tumor mutations in combination with the FOLFIRI regimen colorectal cancer, but data have not yet been published.

### 5.6. CHR-3996

CHR-3996 is a new potent compound in development for clinical trials in solid tumors [125]. In a phase I trial in patients with refractory solid tumors, the recommended phase II dose was set at 40 mg/d orally once daily [126]. The drug was generally well tolerated, with dose limiting toxicities being represented by thrombocytopenia, fatigue, increase of plasma creatinine and atrial fibrillation [126].

## 6. Conclusions

Since the first HDACI was approved for clinical use in 2006, several novel Hb-HDCAIs have been tested in clinical trials, either as single agents or in combination therapies. Although many studies are still ongoing (Table 6), the available results disappointed the high expectancies based on the principle that increased function of HDAC is required for cancer cell survival and proliferation.

**Table 6:** Ongoing phase II clinical trials of Hb-HDACIs in solid tumors [127].

Disease	Compound	Regimen	No. Pts.	End-Point	ClinicalTrials. gov Identifier
Relapsed/refractorysarcomas-age 4–21 years	Vorinostat	*Vorinostat*: orally on a daily × 4 schedule +*Etoposide:* at a fixed dose i.v. daily × 3 days	50	DLT MTD RR	NCT01294670
HER2-positive locally recurrent or metastatic breast cancer	Vorinostat	*Vorinostat*: 300 mg × 4 days on, then 3 days off +*Lapatinib:* 1,250 mg daily	47	CB	NCT01118975
Metastatic RCC	Vorinostat	*Vorinostat*: escalating doses PO BID on days 1–14 *+Bevacizumab*: IV on day 1 of a 21-day cycle	42	MTD PFS	NCT00324870
Advanced soft tissue sarcomas	Vorinostat	*Vorinostat*: orally once daily for 14 days + *Bortezomib*: IV on days 1, 4, 8, 11 of a 21-day cycle	45	RR	NCT00937495
Metastatic NSCLC	Belinostat	*Belinostat*: dose escalation with starting dose 1 g/m^2^ i.v. on days 1-5 of a 21-day cycle +Carboplatin AUC 6 +Paclitaxel 200 mg/m^2^	35	MTD	NCT01310244
Recurrent GBM	Panobinostat	*Panobinostat*: orally three times per week every other week +*Bevacizumab*: i.v. on days 1 and 15 of a 28-day cycle	67	MTD PFS	NCT00859222
Advanced sarcomas	Abexinostat	*Abexinostat:* orally on days 1–5 +*Doxorubicin* on day 4 of a 21-day cycle	47	MTD RR	NCT01027910
K-Ras mutated advanced CRC	Resminostat	*Resminostat:* orally +OLFIRI i.v.	80	MTD PFS	NCT01277406
HCC pretreated with sorafenib	Resminostat	orally	60	PFS	NCT00943449

Abbreviations: CB, clinical benefit rate; CRC, colorectal carcinoma; DLT, dose limiting toxicity; GBM, glioblastoma multiforme; HCC, hepatocellular carcinoma; MBC, metastatic breast cancer; MDT, maximum tolerated dose; NSCLC, non small cell lung cancer; PFS, progression free survival; RCC, renal cell carcinoma; RR, objective response rate.

Generally, HDACIs seem to be more active in hematological malignancies than in solid tumors. The reasons for this discrepancy are unknown. A possible explanation might be related to the pharmacokinetic profile of HDACIs. In fact, these compounds have a relatively short half-life, ranging from 91.6 to 127 minutes when given orally and from 34.7 to 42.4 minutes when administered intravenously [128]. This short half-life might be responsible for higher drug concentrations in plasma than in tumor microenvironment, resulting in a more pronounced HDAC inhibition in blood rather than in tissue cancer cells. Moreover, solid tumors usually show persistent activation of the signal transducer and activator of transcription (STAT) signaling [129], a pathway that has been reported to be associated with resistance to HDCAIs [130].

Altogether, clinical trials evidence indicates that single agent Hb-HDCAIs show a very limited therapeutic activity. However, when combined with other agents, Hb-HDCAIs are able to increase their anti-tumor activity. We have to wait for the conclusion of current studies to estimate the real clinical efficacy of Hb-HDCAIs.
